# Prognostic significance of caspase 8 associated protein 2 (CASP8AP2) in childhood b cell acute lymphoblastic Leukemia

**DOI:** 10.1007/s12672-025-04147-x

**Published:** 2025-12-11

**Authors:** Omar Arafah, Marihame Ashraf, Ahmed Mustafa Abd Elsalam, Sally Elfishawi, Mahmoud Hammad

**Affiliations:** 1 Department of Pediatric Oncology, National Cancer Institute, Cairo University, 1 Fom El Khaleeg Street, Kasr El Aini Avenue, Cairo, 11796 Egypt; 2 Department of Clinical Pathology, National Cancer Institute, Cairo University, 1 Fom El Khaleeg Street, Kasr El Aini Avenue, Cairo, 11796 Egypt

**Keywords:** Childhood acute lymphoblastic leukemia, CASP8AP2 gene, Apoptosis, Chemotherapeutic sensitivity

## Abstract

**Background:**

Improved treatment of childhood acute lymphoblastic leukemia (ALL) depends on the identification of new molecular markers that can predict treatment response and clinical outcome. Examination of the expression patterns of a set of genes at the RNA level is one of these new modalities. The prognostic significance of caspase-8-associated protein 2 (CASP8AP2), an apoptosis-related gene, in pediatric ALL is controversial.

**Methods:**

A prospective study of 70 newly diagnosed ALL patients who were treated in the National Cancer Institute (NCI), Cairo University during the period from 1st of October 2019 till the end of September 2023, to measure the CASP8AP2 expression level in bone marrow samples at the time of diagnosis and at the end of induction therapy using real-time quantitative PCR, and to assess its relation with different prevalent prognostic variables and disease outcome. All cases were followed up till end of December 2024.

**Results:**

Higher initial CASP8AP2 gene expression was associated with hyperdiploid karyotyping (p = 0.009), molecularly favorable mutations (p = 0.002), early induction response (p = 0.025), and low-risk patients (p < 0.001) and had a significant impact on lowering events in the first 3 years of follow-up (p = 0.001). Meanwhile, examining post-induction gene levels failed to show similar results.

**Conclusions:**

Higher initial CASP8AP2 gene expression was associated with favorable impact on event-free survival in pediatric ALL patients. Post-induction levels did not show similar correlation. Future larger studies are needed to confirm the favorable association and to search for other possibly related prognostic factors to further refine risk stratification.

## Introduction

Acute lymphoblastic leukemia (ALL) is the most common cancer in children, comprising around 25% of all childhood malignancies [1]. In the past two decades, the increased cure rates for pediatric ALL have been one of the major achievements in cancer therapy, with the current 5-year survival rate increasing to about 90% in children younger than 15 years, mainly in developed countries [2].

Great advances have been made in risk stratification, more precise response monitoring, treatment plan modification and prediction of prognosis in pediatric ALL [3]. Yet, there are certain groups of patients whose response to treatment and durable remissions are not explained by the routinely used criteria for risk assignment and response assessment tools. Therefore, new prognostic markers should be explored as relapses remain the main obstacle against curing childhood ALL [4].

Cell apoptosis is initiated by extracellular and intracellular signals via two main pathways, the mitochondrial death pathway (the intrinsic pathway) and the death receptor–mediated pathway (the extrinsic pathway) [5]. Defects in the apoptotic process disrupt the delicate balance between cell proliferation and cell death. Studies have shown that leukemic cells adopt these mechanisms to resist chemotherapeutic agents [6].

CASP8AP2, a gene found on chromosome 6q15–16.1, encodes the enzyme needed to help apoptosis in response to chemotherapy [7, 8], as the CASP8AP2 protein interacts with caspase-8 death-effector domain and plays an essential regulatory role in Fas and tumor necrosis factor–mediated apoptosis [9]. CASP8AP2 has been the focus of interest in many research projects. Its prognostic significance in pediatric ALL had been extensively studied before, with inconsistent results [21, 23, 25, 27].

Caspases are subdivided into three groups according to structure and function. Group I (inflammatory caspases-1, -4, -5, -11, and -12), Group II (initiators caspases-2, -8, -9, and -10), and Group III (caspases-3, -6, and -7) [10].

In current study, we aimed to assess the impact of the level of CASP8AP2 gene expression pre- and post-induction therapy on the outcome in pediatric ALL and to analyze its associations with early response to treatment and other prevalent prognostic factors.

## Materials and methods

This prospective cohort study was conducted on 70 newly diagnosed ALL pediatric patients who were treated at the pediatric oncology department, National Cancer Institute (NCI), Cairo University—Egypt from 1 st of October 2019 till the end of September 2023.

Medical records were reviewed for epidemiological data (age groups, sex), date of presentation, clinical data, initial investigations. Bone marrow aspirates (BMA) were assessed for immunophenotyping by flow cytometry, ALL-associated fusion gene transcripts by fluorescence in situ hybridization (FISH), karyotyping (G-banding), along with cerebrospinal fluid (CSF) examination. Risk assignment was done according to St. Jude’s risk stratification [11], and treatment data were recorded (e.g.: date and type of chemotherapy received).

Data of relapse were also collected, including timing of relapse (early or late), type of relapse (hematological, central nervous system [CNS], or combined), salvage chemotherapy given (e.g.: timing and number of cycles), and response to salvage therapy.

Survival analysis data were collected (date of diagnosis, date of relapse, and date of death/last follow-up).

### Eligibility criteria

All included patients in the study aged between 1 and 18 years old, confirmed to have ALL by immunophenotyping, risk assigned and treated according to St. Jude total XV protocol [11], and had a sufficient bone marrow (BM) sample for total RNA/microRNA (miRNA) extraction as identified through the clinical pathology department data registry.

Exclusion criteria: Patients who were included prospectively and died before the end of induction and patients without sufficient material for total RNA extraction at end of induction.

### MRD assessment

At the time of diagnosis, peripheral blood or bone marrow samples were taken; samples were examined the same day [12]. A wide range of monoclonal antibodies, including CD45, CD19, CD10, CD22, CD34, CD79A, CD1PE, CD7, CD2, CD4, CD8, CD16, CD56, CD99, TDT, CD33, CD13, CD11B, CD15, NG2, and CD117, were acquired from Beckman Coulter in Miami, USA, for the purpose of lineage assignment. The monoclonal antibodies used were from Dako (Cytomation, Denmark) and included MPO, CD3, Kappa, Lambda, CD5, cytoplasmic μ, CD20, and MHC class II. 50 μL of a sample with an adjusted cell count of approximately 1 × 10^6^ cells per tube was incubated with monoclonal antibodies according to the manufacturer’s instructions for 30 min at room temperature in the dark, after which the sample was lysed, and excess antibodies were removed with Phosphate Buffered Saline (PBS) [13].

In 500 mL of PBS, the cells were suspended. The analysis was carried out using a Navious cytometer. Firstly, the blast population was selected on the basis of forward scatter versus side scatter and then on the basis of CD45 versus side scatter.

The cut-off value for surface monoclonal antibodies was 20%, while that for cytoplasmic antibodies was 10% [14]. Utilizing a Navious cytometer (Beckman Coulter, Miami, Florida), flow cytometry (FCM) was performed to evaluate MRD.

Using INTA Prep permeabilization reagent from Beckman Coulter, intracellular staining was performed by fixing cells with reagent 1 (formaldehyde fixation reagent), washing, inducing permeability with reagent 2 (using saponin for permeability), and lysing any remaining erythrocytes.

For the detection of MRD, the following monoclonal antibodies were used in four color combinations. BCP-ALL: TdT/CD10/CD19/CD45; CD10/CD20/CD19/CD45; CD34/CD38/CD19/CD45; CD34/CD22/CD19/CD45; CD19/CD34/CD45; CD10/CD20/CD22/CD45. Since this combination was one of the highest incidence marker expressions and helped distinguish hematogones from residual blasts in ALL instances after induction, it was identified at diagnosis and post-induction following morphological remission for MRD tracking.

Cutoff values were deemed to be > 20% for surface monoclonal antibodies, whereas cytoplasmic values were ≥ 10%. The median number of events measured in the assay was 1,250,000 (range: 750,000–1,600,000). The detection limit (LOD) is 0.002% or one in every 50,000 cells. The lower limit of quantification (LLOQ) is 0.01% (1–10,000). Sequential gating was employed. The minimum target sensitivity for quantifying MRD was defined as the ability to detect 30 clustered MRD events in 3 × 10^5^ total cellular events (0.01%).

The cutoff point for MRD1 (D15) was < 10⁻^3^ (0.1%), and for MRD at any time point, it was < 10⁻^4^ (0.01%) [15] at D42 (End of induction), week 17, week 48 and end of treatment in patients who ended chemotherapy.

### CASP8AP2 gene expression analysis

*Nucleic Acid Extraction:* Under strict aseptic conditions, bone marrow samples drawn in two EDTA tubes containing 3 mL each were utilized to extract RNA. Extraction of total cellular RNA from blood was done using QIAamp RNA Blood Mini Kit for total RNA purification (Qiagen, Hilden, Germany). Concentration and purity of RNA was checked by measuring the absorbance at 260 nm (A260) using the Nanodrop spectrophotometer.

*Analysis of CASP8AP2 gene expression:* Following RNA extraction, reverse transcription was performed, and first-strand cDNA was prepared using 1 μg of total RNA using Applied Biosystems™ High-Capacity cDNA Reverse Transcription Kit (Thermo Fisher Scientific, USA). Quantitative RT-PCR assays were performed using the SLAN^®^-48P Real-Time PCR System Hongshi system (Shanghai Hongshi Medical Technology Co., Ltd.) with Taqman Universal Mastermix (Thermo Fisher Scientific, USA) and inventoried Taqman assay (Applied Biosystems, Life Technologies) for CASP8AP2 mRNA (Hs01594281_m1) and ABL1 (Hs01104728_m1) as the housekeeping gene.

A total of 10 pediatric control samples were used for gene expression analysis. These were gathered from normal pediatric bone marrow donor samples supplied as screening samples before donation.

Data from the amplification plot was obtained and analyzed to determine the relative expression of CASP8AP2 gene among patients and control samples and then compare cycle threshold (CT) of the target genes to the CT of the housekeeping gene. To determine the relative expression, we used the comparative cycle threshold (2 − ΔΔCT) method where the data were represented as the fold change in gene expression compared to an endogenous reference gene.

In our study, the endogenous reference gene was ABL1. ΔCT data (CT target gene–CT endogenous reference gene) was used [16].

### Statistical methods

Cut-off for gene expression was estimated at the maximum sensitivity and specificity using the Youden’s Index of the Receiver Operating Characteristics (ROC) curve coordinates. The cut-off values for CASP8AP2 gene expression were used to designate groups at diagnosis, where groups below cutoff threshold were designated low expressors and groups showing gene expression greater or equal to the threshold, were designated high expressors. The association between high and low expression levels and different variables was tested using Pearson’s Chi-squared test and Fisher’s exact test. To compare CASP8AP2 expression levels between different variables, we employed the Wilcoxon and Kruskal–Wallis rank sum tests.

For survival analysis, the Kaplan–Meier method was used and the survival experience compared using the log-rank test. Alpha was two-sided and set at 0.05. All analyses were conducted using R version 4.1.0. A *p*-value less than 0.05 indicated statistical significance.

Overall survival (OS) was defined as the period between the date of diagnosis and the date of death from any cause or the date of the patient’s last follow-up.

Event-free survival (EFS) was defined as the period between the date of diagnosis and the date of relapse or to date of last follow-up for patients without events.

## Results

Initial patients’ characteristics are summarized in Table 1.

**Table 1:** Clinical characteristics and initial laboratory investigations of the whole cohort (N = 70)

Characteristics		N = 70^*1*^	% (%)
Age group	≤ 10 years	52	74
	> 10 years	18	26
Gender	Male	45	64
	Female	25	36
Initial TLC Group	≥ 50	22	31
	< 50	48	69
Initial BMA % Blasts		91 (85–95)	
Immunophenotyping	BCP C-ALL	50	71
	BCP Pre-B ALL	16	23
	BCP Pro-B-ALL	4	5.7
Molecular studies	Negative	43	61
	t (12;21)	15	21
	t (4;11)	4	5.7
	t (9;22)	6	8.6
	t (1;19)	2	2.9
Karyotyping	Hyperdiploidy	17	24
	Hypodiploidy	3	4.3
	Normal	50	71
CNS Status	I	58	83
	III	3	4.3
	TLP without blasts	9	13
D15 BMA	CR	55	79
	Not in CR	15	21
D15 BMA MRD	Negative	55	79
	Positive	15	21
D42 BMA	CR	70	100
D42 BMA MRD	Negative	57	81
	Positive	13	19
Risk Stratification	LR	28	40
	SR	37	53
	HR	5	7.1
Initial BMA Gene Exp		3.3 (1.8–5)	
End of Induction Gene Exp		1.92 (0.98–3.50) *	
Last Chemotherapy	Induction	1	1.4
	Consolidation	2	2.9
	Week 7	27	39
	Week 48	14	20
	Ended treatment	13	19
	FLAG/M	13	19
RELAPSE	YES	13	19
	NO	57	81
Remission Status	Maintained CR	57	81
	Early Isolated Haematological Relapse	7	10
	Late Isolated Haematological Relapse	6	8.6
Status	Alive	57	81
	Dead	13	19

^1^Median (IQR); n (%); * 5 N/A

*TLC* Total leukocytic count, *LR* Low-risk, *SR* Standard-risk, *HR* High-risk, *CNS* Central nervous system, *TLP*: Traumatic lumbar puncture, *ALL* Acute lymphoblastic leukemia

### CASP8AP2 gene expression initially and post induction and its relations to different variables

**Fig. 1:**
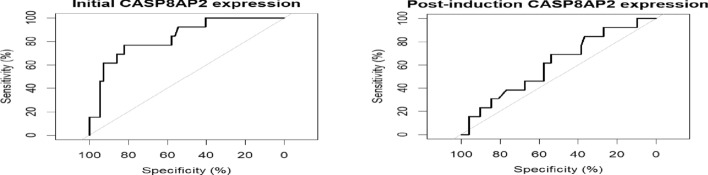
ROC curve for low and high CASP8AP2 gene expression levels

A ROC curve (receiver operating characteristic curve) was used to define levels of low and high expression of CASP8AP2 gene (Table 2) Figure 1.

**Table 2:** CASP8AP2 gene expression cut-off in the initial and post-induction BMA

	Initial BMA	Post-Induction BMA
Threshold	1.95 (1.17–5.6)	1.94 (0.59–4.37)
AUC	83.6% (71.2–95.5%)	61.2% (44.6–77.8%)
Sensitivity	76.9% (53.9–100)	69.2% (23.1–100)
Specificity	82.5% (47.4–98.3)	53.8% (19.2–55.8)
PPV	90.0	42.9
NPV	94.0	87.5

Patients with high initial gene expression levels were more likely to be associated with hyperdiploid karyotyping (p = 0.007), molecular favorable mutations (t (12;21) and negative mutations) (p = 0.038), good early (Day 15) and end-of-induction (Day 42) morphological and MRD response (p = 0.019 and p = 0.029, respectively), and low-risk stratification (p < 0.001). Meanwhile, the post-induction high gene expression levels failed to maintain the same statistically significant correlation with the same favorable variables (Table 3).

**Table 3:** Relation of initial patients’ characteristics and treatment response to the initial and post-induction BMA CASP8AP2 gene expression level

Characteristic	Initial BMA CASP8AP2 Gene Expression level N = 70 Median (IQR)	p	Post-Induction CASP8AP2 Gene Expression level N = 70 Median (IQR)	p
Age group				
> 10 years	2.5 (1.2–4.0)	0.2	1.36 (0.98–2.76)	0.5
≤ 10 years	3.4 (2.0–5.1)		1.98 (0.99–3.97)	
TLC group				
< 50,000	3.50 (2.10–5.13)	0.066	1.98 (0.96–3.74)	0.7
≥ 50,000	2.40 (1.00–4.30)		1.49 (1.00–2.28)	
Karyotyping				
Hyperdiploid	4.70 (2.30–5.30)	0.007*	2.94 (1.98–5.22)	0.09
Hypodiploid	0.50 (0.40–0.80)		1.11 (0.67–4.44)	
Normal	3.10 (1.70–4.60)		1.62 (0.96–2.36)	
Molecular				
Negative	3.80 (2.00–5.10)	0.038*	2.07 (1.00–4.05)	0.3
t (12;21)	3.50 (2.30–5.40)		1.94 (1.00–2.56)	
t (1;19)	2.70 (2.00–4.70)		4.72 (3.35–6.08)	
t (4;11)	5.20 (3.00–7.40)		1.18 (0.92–1.42)	
t (9;22)	1.60 (0.70–1.90)		1.30 (0.71–2.00)	
D15 BMA		0.019*		0.7
CR	3.5 (2.20–5.80)		1.98 (0.99–3.72)	
Not in CR	1.8 (1.40–3.40)		1.46 (0.89–2.07)	
End of induction MRD		0.292		0.7
Positive	2.3 (1.40–3.20)		1.96 (0.98–3.50)	
Negative	3.8 (1.90–5.30)		1.46 (1.00–2.53)	
Risk stratification		< 0.001*		0.3
High risk	0.90 (0.50–1.00)		1.11 (0.98–1.81)	
Low risk	4.80 (3.30–6.80)		2.30 (0.98–3.94)	
Standard risk	2.50 (1.40–4.00)		1.77 (0.99–3.09)	

Moreover, we calculated the high and low CASP8AP2 gene expression levels within each variable including the above proven associated favorable ones. Significantly high pre-induction CASP8AP2 gene levels compared to lesser low levels were mostly encountered in patients with hyperdiploid karyotyping (p = 0.009), molecular favorable (p = 0.002), early induction response (p = 0.025), and low-risk patients (p < 0.001). This difference in expression levels within each variable did not persist in post-induction gene levels (Table 4).

**Table 4:** Relation of different CASP8AP2 gene expression cut-off levels in the initial pre-induction and post-induction BMA to the different prognostic features

Characteristic	Initial bone marrow CASP8AP2 cut-off levels			Post induction bone marrow CASP8AP2 cut-off levels		
	high, N = 50^*1*^	low, N = 20^*1*^	*p*-value^*2*^	high, N = 32^*1*^	low, N = 33^*1*^	*p*-value^*2*^
Age Group			0.3			0.11
> 10 years	11 (22%)	7 (35%)		6 (19%)	12 (36%)	
≤ 10 years	39 (78%)	13 (65%)		26 (81%)	21 (64%)	
TLC Group			0.12			0.14
< 50,000	37 (74%)	11 (55%)		24 (75%)	19 (58%)	
≥ 50,000	13 (26%)	9 (45%)		8 (25%)	14 (42%)	
Karyotyping			0.009			0.076
Hyperdiploid	15 (30%)	2 (10%)		11 (34%)	4 (12%)	
Hypodiploid	0 (0%)	3 (15%)		1 (3.1%)	2 (6.1%)	
Normal	35 (70%)	15 (75%)		20 (63%)	27 (82%)	
Molecular			0.002			0.2
Negative	32 (64%)	11 (55%)		21 (66%)	18 (55%)	
t (1;19)	2 (4.0%)	0 (0%)		2 (6.3%)	0 (0%)	
t (12;21)	12 (24%)	3 (15%)		7 (22%)	7 (21%)	
t (4;11)	4 (8.0%)	0 (0%)		0 (0%)	4 (12%)	
t (9;22)	0 (0%)	6 (30%)		2 (6.3%)	4 (12%)	
D15 BMA			0.025			0.3
CR	43 (86%)	12 (60%)		27 (84%)	24 (73%)	
Not CR	7 (14%)	8 (40%)		5 (16%)	9 (27%)	
End of Induction MRD			0.5			0.6
Negative	42 (84%)	15 (75%)		27 (84%)	26 (79%)	
Positive	8 (16%)	5 (25%)		5 (16%)	7 (21%)	
Relapse	3 (6.0%)	10 (50%)	< 0.001	4 (13%)	9 (27%)	0.14
Risk Stratification			< 0.001			0.3
High Risk	0 (0%)	5 (25%)		1 (3.1%)	4 (12%)	
Low Risk	24 (48%)	4 (20%)		14 (44%)	10 (30%)	
Standard Risk	26 (52%)	11 (55%)		17 (53%)	19 (58%)	

^1^n (%)

^2^Pearson’s Chi-squared tests; Fisher’s exact test

### Survival outcome compared to gene expression levels and cause specific mortalities

**Fig. 2:**
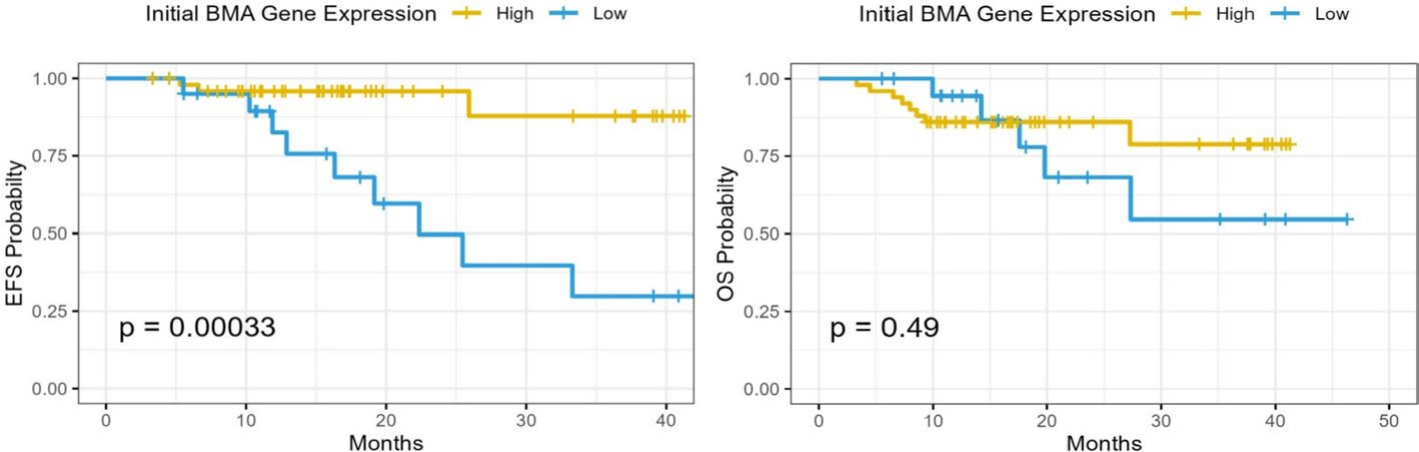
Event-free and Overall survival analysis according to the expression level of CASP8AP2 gene in pre-induction BMA

**Fig. 3:**
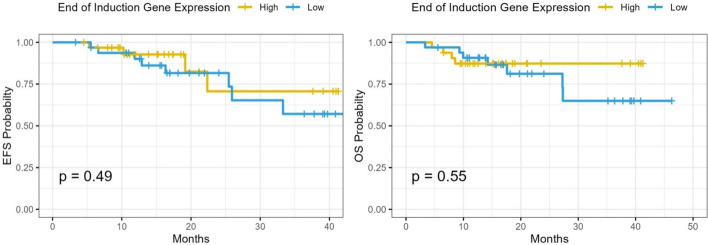
Event-free and Overall survival analysis according to the expression level of CASP8AP2 gene in post-induction BMA

Median follow-up time was 1.5 years (95% CI 1.3–1.8). Survival outcome was re-analyzed after dividing patients by ROC curve into high and low gene expression levels. Within the first three years of follow-up, high gene expression levels in the pre-induction BMA had been associated with better survival in terms of fewer events (*p*-value < 0.001) Fig. 2**.** This contrasts with post induction BMA gene level which did not correlate with lesser events (*p*-value 0.49**) **Fig. 3.

Out of the 70 patients included in the study, there were 13 mortality cases (19% of the whole cohort). Five patients (39%) died in the first year, seven (54%) in the second year, and one (7%) in the third year after induction therapy, respectively.

In 4 out of the 5 patients who died in the first year, deaths occurred because of septic complications during maintenance therapy and all of them exhibited a high initial gene expression level. Only one patient had encountered early isolated hematological relapse and died from septic complications following intensive salvage chemotherapy. This patient had a low initial gene expression level.

Among the seven patients who died during the second year, two died from septic complications during maintenance therapy, and one of them had a low initial gene expression level. The remaining five patients experienced relapse: two died from septic complications after receiving intensive salvage chemotherapy, and three died from refractory disease (palliative cases). Of the five relapsed cases, four exhibited a low initial gene expression level.

Only one patient died during the third year, following septic complications after intensive salvage chemotherapy for an isolated hematological relapse. This patient exhibited a low initial gene expression level.

To nullify the possible theoretical confounding effects of known prognostic factors that have been previously shown to influence survival outcomes, either favorable or unfavorable, survival outcome was re-analyzed during the first year of follow-up in relation to the level of gene expression at two points (initial and post-induction), within the same risk variable subgroups. Within patients younger than 10 years old, showing good early induction response and low-risk patients, those with high initial CASP8AP2 gene expression levels showed the lowest events (p = 0.001, < 0.001, < 0.001 respectively). This was not found in high post induction CASP8AP2 gene expression levels within the same groups (Table 5).

**Table 5:** First year EFS in high and low CASP8AP2 gene expression levels in comparison to different age groups, early response D15 and risk stratification

Characteristic	EFS 12 Months	*p*-value^*1*^	Characteristic	EFS 12 Months	*p*-value^*1*^	Characteristic	EFS 12 Months	*p*-value^*1*^
1–10 years (N = 52)			IN CR D15 (N = 55)			High/Standard risk (N = 42)		
Initial BMA Gene Expression		0.001	Initial BMA Gene Expression		< 0.001	Initial BMA Gene Expression		0.1
High	95% (88–100%)		High	98% (93–100%)		High	92% (82–100%)	
Low	89% (71–100%)		Low	79% (56–100%)		Low	87% (71–100%)	
End of Induction Gene Expression		0.5	End of Induction Gene Expression		0.5	End of Induction Gene Expression		0.7
High	96% (89–100%)		High	95% (86–100%)		High	88% (74–100%)	
Low	90% (77–100%)		Low	90% (79–100%)		Low	91% (79–100%)	
11–19 years (N = 18)			NOT IN CR D15 (N = 15)			Low risk (N = 28)		
Initial BMA Gene Expression		0.057	Initial BMA Gene Expression		0.6	Initial BMA Gene Expression		< 0.001
High	100% (100–100%)		High	86% (63–100%)		High	100% (100–100%)	
Low	71% (45–100%)		Low	88% (67–100%)		Low	75% (43–100%)	
End of Induction Gene Expression		0.9	End of Induction Gene Expression		> 0.9	End of Induction Gene Expression		0.8
High	83% (58–100%)		High	80% (52–100%)		High	100% (100–100%)	
Low	91% (75–100%)		Low	89% (71–100%)		Low	89% (71–100%)	

### Multivariable analysis

To assess whether the effect of gene expression level alone, independent of other prognostic variables, was associated with the outcome; subgroup analysis was performed within homogeneous risk groups. Among patients ≤ 10 years, with early induction response and low-risk classification, those with high initial CASP8AP2 gene expression had the lowest number of events (p = 0.001, < 0.001, < 0.001, respectively). These associations were not maintained with post-induction gene expression.

Multivariable Cox regression yielded an EFS hazard ratio (HR) of 0.33 (95% CI 0.08–1.45, p = 0.14) for high initial CASP8AP2 expression. The model was overfitted with an observed: expected ratio = 4.9 at 3 years and EFS was significantly correlated with last follow MRD. Caution is warranted in interpretation due to small sample size, event count, and wide error margins (Table 6 and Table 7).

**Table 6:** Event-Free Survival (EFS): Uni- and Multivariable Cox Regression Analysis

Variable	Univariable HR (95% CI)	p	Multivariable HR (95% CI)	p
Age ≤ 10 versus > 10	1.15 (0.31–4.25)	0.8	–	–
TLC ≥ 50 versus < 50	1.38 (0.43–4.39)	0.6	–	–
Karyotyping				
Hypodiploid versus Normal	24.7 (5.12–119)	< 0.001	–	–
Hyperdiploid versus Normal	1.26 (0.26–6.13)	0.8	–	–
D15 BMA (Not CR vs. CR)	0.99 (0.27–3.67)	0.9	–	–
End Induction MRD				
Positive versus Negative	2.00 (0.60–6.67)	0.3	–	–
Last Follow-up MRD				
Positive versus Negative	34.9 (7.47–163)	< 0.001	23.9 (4.68–122.0)	< 0.001
Risk Stratification				
HR	2.68 (0.49–14.8)	0.3	–	–
CASP8AP2 Initial (Low vs. High)	0.33 (0.08–1.45)	0.14	–	–

**Table 7:** Overall Survival (OS): Uni- and Multivariable Cox Regression Analysis

Variable	Univariable HR (95% CI)	p	Multivariable HR (95% CI)	p
Age ≤ 10 versus > 10	2.02 (0.45–9.13)	0.3	–	–
TLC ≥ 50 versus < 50	0.91 (0.28–2.98)	0.9	–	–
Karyotyping				
Hypodiploid versus Normal	6.05 (1.22–30.1)	0.028	–	–
D15 BMA (Not CR vs. CR)	1.36 (0.42–4.43)	0.6	–	–
End Induction MRD				
Positive versus Negative	2.50 (0.87–7.26)	0.1	–	–
Last Follow-up MRD				
Positive versus Negative	7.92 (2.64–23.8)	< 0.001	3.18 (1.38–6.40)	< 0.01
Risk Stratification				
HR	1.66 (0.83–3.26)	0.2	–	–
CASP8AP2 Initial (Low vs. High)	0.69 (0.20–2.36)	0.56	–	–

## Discussion

Outcomes of pediatric ALL have improved remarkably during the last five decades. Such improvements were made possible by the incorporation of new diagnostic technologies, the effective administration of conventional chemotherapeutic agents, and the provision of better supportive care [17]. Despite this success, accurately predicting relapse is still a challenge [18], emphasizing the need to explore and study further potential target effectors more deeply than before, trying to find out their possible roles and contribution.

Studies have identified that alterations in the baseline level of expression of genes controlling the cell cycle, DNA repair, and apoptosis may participate in disordered leukemic cell proliferation and accordingly affect the drug response and clinical outcome of leukemia patients [19]. Identification of such new gene markers is important to implement enhancements to disease classification systems and to productively target disease with novel therapies [20].

Many studies have reported the importance of the initial gene level of CASP8AP2, an apoptosis-related gene, for the optimal response to chemotherapy and maintaining continuous complete remission in pediatric ALL, as concluded by Flotho et al., Jiao et al., Jin et al., Juarez-Velazquez et al., Liu et al., and Remke et al. [21–27]. However, others, like Kang et al. [28] and Yang et al. [29], failed to prove similar relationships.

In this study, we started first to prospectively investigate whether the initial CASP8AP2 gene expression level has a certain predilection to different epidemiological, laboratory, molecular, response, and risk grouping factors. Patients with hyperdiploid karyotyping (p = 0.007), molecularly favorable mutations (p = 0.038), good morphological and molecular responses on day 15 and day 42 of induction therapy (p = 0.019 and p = 0.029, respectively), and low-risk stratification (p < 0.001) were positively associated with higher initial gene expression levels. Based on our results, the post-induction gene expression levels failed to maintain the initial statistically significant correlation with the above favorable variables as was expected based on the presumed mechanism of action.

Analysis of these associations represents a double-edged sword. While the conclusion supports the link between higher gene levels and the known favorable variables and consequently a better response; it also raises the possibility that other favorable confounding variables may be responsible for the positive outcomes observed in these patients.

Concluding a definitive positive effect of high CASP8AP2 gene level on survival is not easy due to the confounding effect of other known favorable risk variables. Thus, a ROC curve was proposed to define levels of low and high expressions of CASP8AP2 gene.

The ROC coordinates were estimated using the empirical trapezoidal method (Delong) with the {pROC} package v1.18.5. Survival data were then re-examined and as in Flohr et al., fewer events in the first 3 years of follow-up were observed with initial higher gene expression [21]. The pre-induction higher expression levels were as expected encountered in hyperdiploid, molecularly favorable, early induction responder and low risk patients, but similarly these higher expressions did not persist with the same risk variables in post induction levels. Losing the initial higher gene expression favorable effect on survival post-induction chemotherapy and its associations with favorable variables is not well understood and recalls the previous argument about the independent effect of the CASP8AP2 gene. Whether this is related to decreased blast cell number carrying the CASP8AP2 gene because of chemotherapy needs to be confirmed in future studies.

To nullify the possible theoretical confounding effect of some known previously proven favorable or unfavorable prognostic factors and to confirm if the level of gene expression independently can be related to the effect on survival, the incidence of events that occurred during the first year of follow up in high and low CASP8AP2 gene expression level was re-examined at two points (initially and post-induction) within the same risk variable groups. As expected, within patients younger than 10 years, early induction response, and in low-risk patients, those with high initial CASP8AP2 gene expression levels showed the lowest events, but this again was not found in high post-induction gene level expressions in any comparable way.

This emphasizes that the post-induction gene level has no prognostic value in pediatric patients with ALL. Moreover, within the same older age, standard/high risk patients and those not in remission, the higher initial CASP8AP2 gene level did not positively impact the survival in those groups, denoting that other factors might counteract its favorable effect in a significant way.

High CASP8AP2 gene expression is noticed in rapidly proliferating leukemic cells allowing better response to chemotherapeutic agents [30]. This is the best encountered scenario before induction of disease remission and this could partially explain why its level and effect get lower after induction of remission, as almost few to non-proliferating leukemic cells are still present.

## Conclusion

Current study showed that higher initial CASP8AP2 gene expression is associated with many known favorable risk factors and has a significant impact on lowering events in the first 3 years of follow-up. On the contrary, examining post-induction gene levels failed to maintain similar statistical results. Adding an initial CASP8AP2 gene-level investigation to the routine diagnostic panel on a larger scale could help prove its independence. Based on our results, measuring post-induction levels is not helpful for the time being and is not recommended. Further future studies investigating a possible role of CASP8AP2 gene expression levels and relapse are recommended.

## Data Availability

The datasets used and/or analyzed during this study are available from the corresponding author on reasonable request.
